# Welcome to *Psychiatry and Clinical Neurosciences Reports*

**DOI:** 10.1002/pcn5.3

**Published:** 2022-01-24

**Authors:** Michio Suzuki, Toshihiko Kinoshita, Nakao Iwata

**Affiliations:** ^1^ Department of Neuropsychiatry University of Toyama Graduate School of Medicine and Pharmaceutical Sciences Toyama Japan; ^2^ Department of Psychiatry Kansai Medical University Osaka Moriguchi Japan; ^3^ Department of Psychiatry Fujita Health University Toyoake Aichi Japan

**Keywords:** Clinical, Neurosciences, Psychiatry

We are very delighted to introduce the first issue of *Psychiatry and Clinical Neurosciences Reports* (*PCN Reports*), the new‐generation, peer‐reviewed, fully online open‐access journal of the Japanese Society of Psychiatry and Neurology. As a companion journal of *Psychiatry and Clinical Neurosciences* (*PCN*), *PCN Reports* addresses the substantial increase in high‐quality research in psychiatry. As *PCN* is able to publish only a limited proportion of high‐quality submissions, *PCN Reports* provides another outlet for researchers and clinicians to publish their precious work. To maximize its scientific impact, *PCN Reports* will maintain high quality by the concerted efforts of skilled national and international editorial board members and dedicated researchers worldwide to conduct peer review.

*PCN Reports* has many advantages as a fully online open‐access journal. It provides more space to publish many scientifically meaningful papers. After rapid peer reviews and decisions, each accepted manuscript will be published, in order, continuously all year round. For authors, more rapid publication, wider circulation, greater visibility, and more citations are expected. For readers, new information comes more rapidly without subscribing to specific journals, and easier access is guaranteed for readers even from economically underdeveloped countries in the world.

*PCN Reports* will publish high‐quality original articles in all fields of psychiatry and clinical neurosciences. It offers publication of specialist research as well as articles on clinical practice with a high standard of publication ethics. Submissions of pilot or feasibility studies for potentially significant advances and of study protocols for ongoing or proposed studies will also be considered for publication. Research fields covered by the scope of *PCN Reports* include molecular psychiatry and psychobiology, clinical neurophysiology and neuropsychology, neuroimaging, neuropsychopharmacology, psychotherapy and psychopathology, social psychiatry and epidemiology, child and adolescent psychiatry, psychogeriatrics and old age psychiatry, and general topics in psychiatry and related fields. *PCN Reports* welcome submissions of review articles summarizing the state‐of‐the‐art findings on a relevant topic or presenting a unique perspective that stimulates further discussions for scientific progress. Moreover, *PCN Reports* will consider well‐documented original case reports addressing findings that have an impact on clinical practice or that shed new light on pathogenesis.

We hope that diverse authors and readers fully enjoy the opportunities newly opened up by *PCN Reports*. Let's keep our fingers crossed for *PCN Reports* sailing with a fair wind. *Bon voyage*!

